# Supplementary material for: Identification and Characterization of Antioxidant Enzyme Genes in Parasitoid Aphelinus asychis (Hymenoptera: Aphelinidae) and Expression Profiling Analysis under Temperature Stress
Source: Insects. 2022 May 9;13(5):447. doi: 10.3390/insects13050447 (PMC9148002; doi:10.3390/insects13050447)
Supplement: Supplementary file 1 [file insects-13-00447-s001.zip › insects-1693026-supplementary.pdf]

Table S1. Gene-specific primers for q-RT-PCR used in this study

Gene name	Forward primer (5'-3')	Reverse primer (5'-3')
AasyGST1	TTGCCGCAGCCATTGGTGTC	GTAGGGACTGTGTGTTGTGGGTTC
AasyGST2	CGCCGAACACATCCGCTACG	GCTATCTGTTGCCCGTTCACCTC
AasyGST3	CCAAGTCAAGGCTCCACCTA	ACTTGCCGCCTACCACTATG
AasyGST4	CGTCGCATTCTTGGGTAAGC	GCGGCAAGATCACGAGAATG
AasyGST5	TCGCTGACATTCTTGGAGCTTGTG	GCACTAAGATTGGGTCGTCCATCC
AasyCAT1	GCGACGGATTCATGGCAATTCAC	GCGACTTAACGGCAGGACACTC
AasyPOD1	CGCTCCACGACCTTACCTTCTTTG	CGGAGACGATACCCACCCACTATG
AasyPOD2	ATAATGACAGCGACATGGCCCTTG	GGACACCGACTGGAGCAAAGC
AasyPOD3	AGAGCAGCGAGAGGAAGGGATG	CGGTGGAGGAGGCGAGGTTC
AasyPOD4	CGCTCCACGACCTTACCTTCTTTG	CGGAGACGATACCCACCCACTATG
AasyPOD5	GTGTTCAACGGGGCGACCAAG	TCCACGACGAAGCCGACGAG
AasyPOD6	GGCACGCAATAGTCCTCCTTCG	CCTACCGAGCAGATGAACCAAGTG
AasySOD1	CACGATGCCAATAACACCACAAGC	CATGCGGAGATGGATGACCTTGG
AasySOD2	CCAGGTGGAAGAAGCGATGT	CACCATCTGCAGGATTCCCA
Aasy18s RNA	GGGAATCGTATCCGTGGACC	GCTCGTGGGTCGATGAAGAA

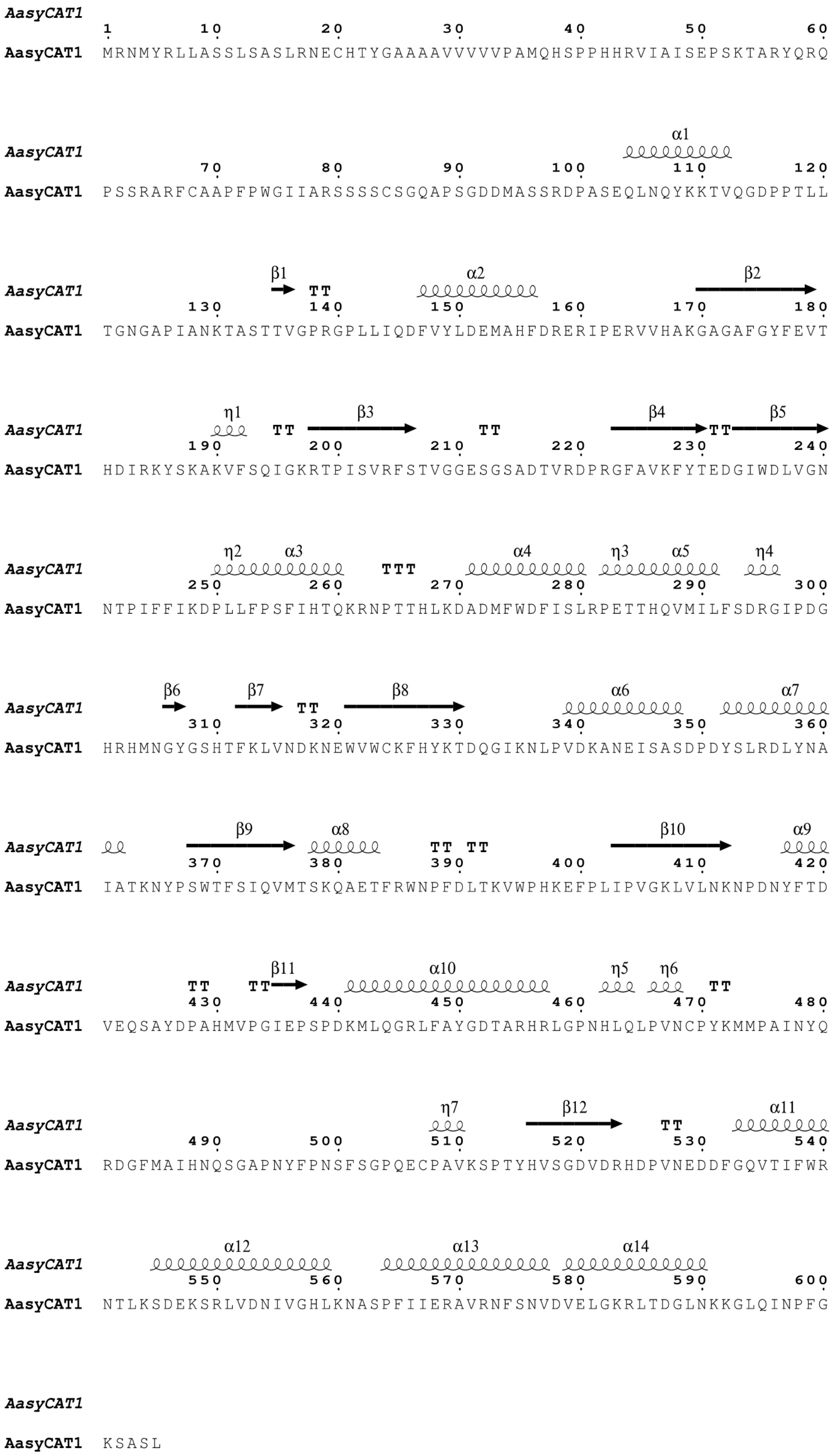

Figure S1. The sequence alignment of *Aphelinus asychis* CAT family. The secondary structures of AasyCAT proteins are shown above the alignment. α-helices and β-sheets were represented by wavy lines and arrows, respectively.

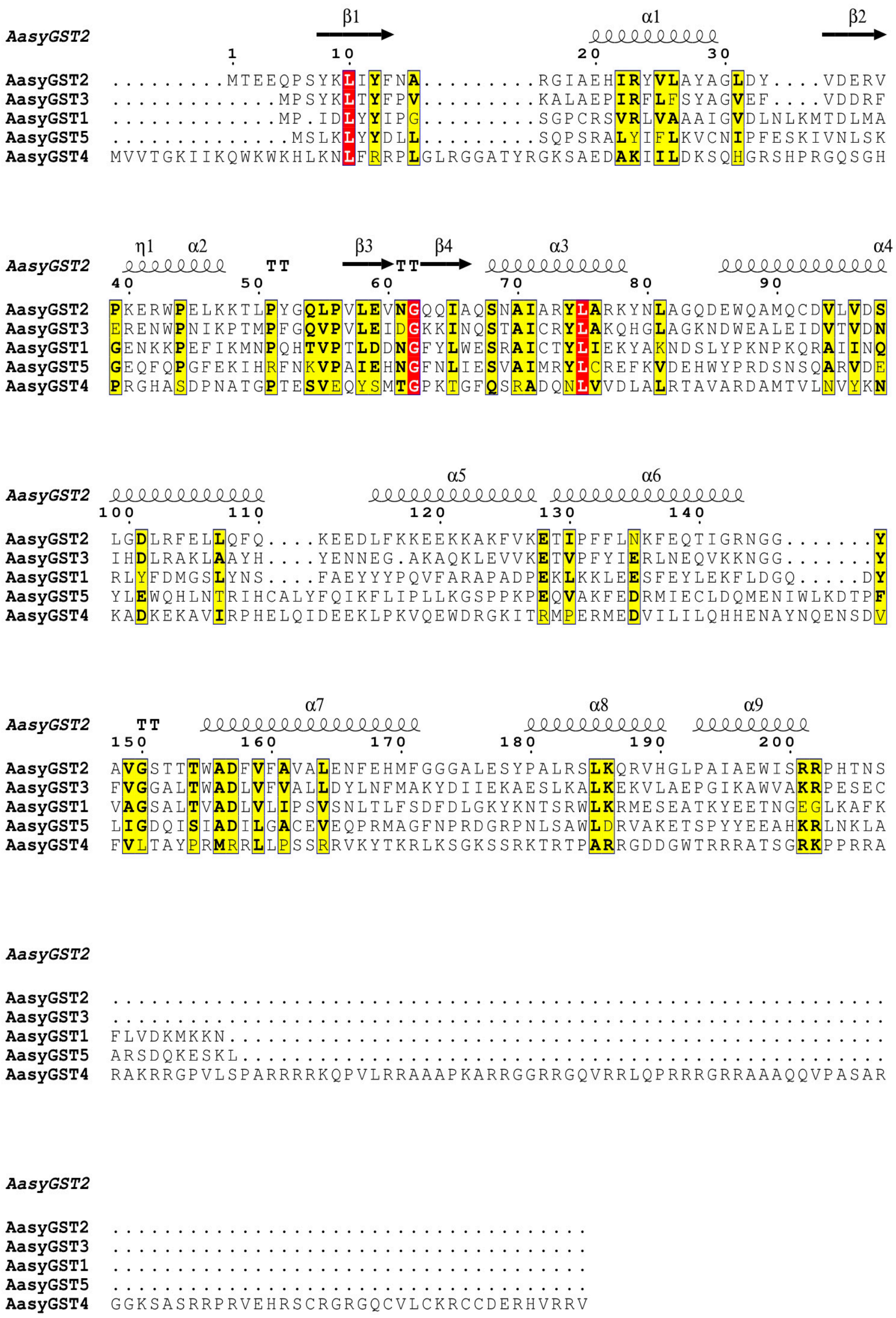

Figure S2. Multiple sequence alignments of *Aphelinus asychis* GST family. The secondary structures of AasyGST proteins are shown above the alignment. α-helices and β-sheets were represented by wavy lines and arrows, respectively.

```
AasyPOD1

AasyPOD1  ............................................................
AasyPOD4  ............................................................
AasyPOD2  ............................................................
AasyPOD5  ............................................................
AasyPOD3  ............MARRSRIVRGLLLAALVAVASAEEPRKKAQRRSSIGDPDDSAVEEARF
AasyPOD6  MWIFYWLRGQIYFYRSAGGVMAGSVATLAVLCVFLQTMGLFGQQNAIGAYKLPFALENYF


AasyPOD1

AasyPOD1  ............................................................
AasyPOD4  ............................................................
AasyPOD2  ............................................................
AasyPOD5  ............................................................
AasyPOD3  DYFN.YHPAPAQKPHPFLSLLYHQTQPAYVVYPQSTSGQPPRWTSSR.............
AasyPOD6  SSNKGYVWEPSQSLVVDTGVRAGKRLPAFDVAVLNASVNFATGVLSRTQRVEASLASSAL


AasyPOD1

AasyPOD1  ............................................................
AasyPOD4  ............................................................
AasyPOD2  ............................................................
AasyPOD5  ............................................................
AasyPOD3  ............................................................
AasyPOD6  YAKARKRLANRQLDDAALEKAQDALVAVGASAYLSQANCVRFGLSVVDCAKYISTMRLEN


                                                     η1      η2   β1     β2  η3
AasyPOD1                                                             TT
                                       1        10        20        30
AasyPOD1  .............................MSGNDDYKSAKSVYDFVVKSIKGEDVPMEKY
AasyPOD4  ......MRLLRSTLFPVGLQQVVRSLSVTMSGNDDYKSAKSVYDFVVKSIKGEDVPMEKY
AasyPOD2  .......MMRLTIALSLLCIASVLGDDSKFNQDTDWSKATSIYDFHAKDIHGKDVSLNKY
AasyPOD5  ...........MISWASIGALLMVGLVAALDQESDWSRASSVYEFHAVDLDGRDVSLDKY
AasyPOD3  GPAPPSCGQYTGPQCRAKSRYRSYDGSCNNLRSPSWGMANTKYARLAPAIYSDGVHAPPV
AasyPOD6  TPLGSSCAAEQQADCDSNSEYRSLDGSCNNVQNPKWGSAFTAYNRILFPQYADGIQMPRK


                                                          β3
AasyPOD1           ...................................................
                 40
AasyPOD1  KGHVLLIVN...................................................
AasyPOD4  KGHVLLIVN...................................................
AasyPOD2  KGHVAVIIN...................................................
AasyPOD5  RGNVLVVFN...................................................
AasyPOD3  ARSGNQLPNSRMVSFALFPDVDVQDPTWSLVAMQWGQFITHDMAMIDG.TTQSKAHATQC
AasyPOD6  ERGVHALPNARAVSVGLASRSARSDVSRTLALVQWSELVSHDLAHTSARKMVWSGRPIVC


                                                                   α1
AasyPOD1  ............................................
                                                              50
AasyPOD1  ............................................VASK.CGLTATNYKEL
AasyPOD4  ............................................VASK.CGLTATNYKEL
AasyPOD2  ............................................VASN.CGLTEKNYKQL
AasyPOD5  ............................................GATKGCPVSSKGFKLL
AasyPOD3  CSDGGQLIEEALSSPLCFPILIPPNDPVYTYERQQCRNFVRSTTDLDRGCTSRYQPAEQL
AasyPOD6  CNEDGQWPLPRYIHPDCNAVSVAENDPDYRKHNVRCLDYVRSLPVLNSDCT..FGPTEQM


                           β4                                  α2
AasyPOD1                                      .........................
              60        70        80        90
AasyPOD1  NELHDKYAESKGLRILAFPCNQFNGQEPGGPEEIC.........................
AasyPOD4  NELHDKYAESKGLRILAFPCNQFNGQEPGGPEEIC.........................
AasyPOD2  QALYEKYGESKGLRVLAFPSNEFAGQEPGTSKEIE.........................
AasyPOD5  QALQERLGPD.GLSLVGFVVDGLTK.ESGSSDESR.........................
AasyPOD3  TVVSGFLDLSLIYGSSDQVAAGLRTGIGGRLITDVRGNREWLPKATNASASCDVFGETDV
AasyPOD6  NQVSHFLDGSTIYGSTLAASSEIRAYEGGLLRANVINGNEYLPVAQAETISQCDS...DN


                                β5              α3
AasyPOD1  .............                          TT                     T
                               100       110       120       130
AasyPOD1  .............SFAERKSVKFDLFEKIDVNGDDTHPLWKYLK..KEKGGTLGSFIKWN
AasyPOD4  .............SFAERKSVKFDLFEKIDVNGDDTHPLWKYLK..KEKGGT........
AasyPOD2  .............EFVKKFNVTFDMYEKIKVNGDGAHPLYKWLKSQKEGEGLITDGIKWN
AasyPOD5  .............QYLDSINVTFDMYAKIRGDGDGAHPLYKWIKSQLPADDNKIPV....
AasyPOD3  CYASGDVRVNQNPQLTILHLLMHREHNRIATQLARINPHWSDETIFQETRRITIAINQQI
AasyPOD6  CYLTGDERANAEPQLAALHTIWMREHNRVARKLAGMIPTWSDETVYQEARRIVTAEIQHI
```

```
                            β6              β7          η4    α4    η5
AasyPOD1  ...............T  ====>  TT       ====>  TT   ~~~~~~~~~~~~~~~~
                            140             150         160

AasyPOD1  ...............FTKFIVDKEGKVVERHGPNVDPSKLSENLEKYF............
AasyPOD4  ............................................................
AasyPOD2  ...............FTKFLISKEGKVVDRFAPTTDPDSMEDSIVKQF............
AasyPOD5  ............................................................
AasyPOD3  SYYEWLPIFIGAENSLNRRIIFKSQGWVNDYDPNVDPSVINEHSNAAFRYFHSLIAGKLL
AasyPOD6  TYKEWLPLLIGKRFANSVGLGVASNYSEVAYQPYIDPAVSNEVATAALRFPSSLKHTTFS
```

```
AasyPOD1

AasyPOD1  ............................................................
AasyPOD4  ............................................................
AasyPOD2  ............................................................
AasyPOD5  ............................................................
AasyPOD3  LVDEQRYSYSYNALRLSDHFNRPGVIEEDGNMDKLTRGMAFQPQEASDQWFDKEITNYLF
AasyPOD6  MTDDDRKIN..DSRRLSDYFYKPRSIESNNVLDGLIRGLATRTAQKMDLHQVVDITNELY
```

```
AasyPOD1

AasyPOD1  ............................................................
AasyPOD4  ............................................................
AasyPOD2  ............................................................
AasyPOD5  ............................................................
AasyPOD3  RAHHRLGDDLRAIDIQRNRDHGLASYNELRVLAGLPKAVEWADFGDLISAENIQKLSQLY
AasyPOD6  KANGAMNLDQISLDIQRGRDHGLPGYNDYRKHCGLRAAKSFDDFLDYVPVEMVKKWRSLY
```

```
AasyPOD1

AasyPOD1  ............................................................
AasyPOD4  ............................................................
AasyPOD2  ............................................................
AasyPOD5  ............................................................
AasyPOD3  ETPDDVDLTVGGSLERHVPGTLAGPTFLNILSEQFWRTRVGDRFWFETGNPEIAFTIEQL
AasyPOD6  KSTDDVDLVIGGMAERPIEDAMLGPTFRCLISSQFLRTRKSDRFFYDSSDQPKPFTLSQL
```

```
AasyPOD1

AasyPOD1  ............................................................
AasyPOD4  ............................................................
AasyPOD2  ............................................................
AasyPOD5  ............................................................
AasyPOD3  SEIRKASISRLMCDNGDNIQLMQRRGFEQVSQSNPLIRCEDIPAIDLSLWRDIQNPGVAS
AasyPOD6  NSLKKVTLARVFCDNGDNIEKMQPNVFLKLQAGNEMRACTDFEAIPS........IDLFA
```

```
AasyPOD1

AasyPOD1  ...........
AasyPOD4  ...........
AasyPOD2  ...........
AasyPOD5  ...........
AasyPOD3  WSTPVSPWFKK
AasyPOD6  WAEKAKAYR..
```

Figure S3. Multiple sequence alignments of *Aphelinus asychis* POD family. The secondary structures of AasyPOD proteins are shown above the alignment. α-helices and β-sheets were represented by wavy lines and arrows, respectively.

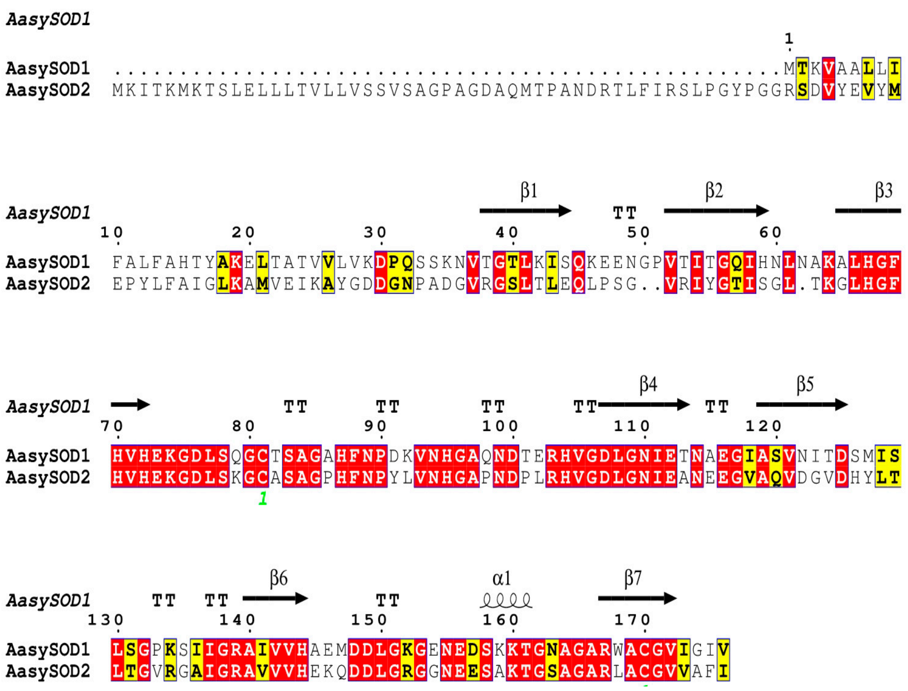

Figure S4. Multiple sequence alignments of *Aphelinus asychis* SOD family. The secondary structures of AasySOD proteins are shown above the alignment. α-helices and β-sheets were represented by wavy lines and arrows, respectively.
